# Estimating the risk of malignancy of adnexal masses: validation of the ADNEX model in the hands of nonexpert ultrasonographers in a gynaecological oncology centre in China

**DOI:** 10.1186/s13048-021-00922-w

**Published:** 2021-12-02

**Authors:** Ping He, Jing-jing Wang, Wei Duan, Chao Song, Yu Yang, Qing-qing Wu

**Affiliations:** 1 Department of Ultrasound, Beijing Obstetrics and Gynecology Hospital, Capital Medical University, 251 Yaojiayuan Road, Chaoyang District, Beijing, 100026 P.R. China; 2 Beijing Maternal and Child Health Care Hospital, 251 Yaojiayuan Road, Chaoyang District, Beijing, 100026 P.R. China; 3 Department of Gynecologic Oncology, Beijing Obstetrics and Gynecology Hospital, Capital Medical University, Beijing, P.R. China; 4 Capacity Building and Continuing Education Center, National Health Commission, Beijing, P.R. China

**Keywords:** ADNEX model, CA 125, Diagnosis, Ovarian tumour, Ultrasonography

## Abstract

**Background:**

This study aims to validate the diagnostic accuracy of the International Ovarian Tumor Analysis (IOTA) the Assessment of Different NEoplasias in the adneXa (ADNEX) model in the preoperative diagnosis of adnexal masses in the hands of nonexpert ultrasonographers in a gynaecological oncology centre in China.

**Methods:**

This was a single oncology centre, retrospective diagnostic accuracy study of 620 patients. All patients underwent surgery, and the histopathological diagnosis was used as a reference standard. The masses were divided into five types according to the ADNEX model: benign ovarian tumours, borderline ovarian tumours (BOTs), stage I ovarian cancer (OC), stage II-IV OC and ovarian metastasis. Receiver operating characteristic (ROC) curve analysis was used to evaluate the ability of the ADNEX model to classify tumours into different histological types with and without cancer antigen 125 (CA 125) results.

**Results:**

Of the 620 women, 402 (64.8%) had a benign ovarian tumour and 218 (35.2%) had a malignant ovarian tumour, including 86 (13.9%) with BOT, 75 (12.1%) with stage I OC, 53 (8.5%) with stage II-IV OC and 4 (0.6%) with ovarian metastasis. The AUC of the model to differentiate benign and malignant adnexal masses was 0.97 (95% CI, 0.96–0.98). Performance was excellent for the discrimination between benign and stage II-IV OC and between benign and ovarian metastasis, with AUCs of 0.99 (95% CI, 0.99–1.00) and 0.99 (95% CI, 0.98–1.00), respectively. The model was less effective at distinguishing between BOT and stage I OC and between BOT and ovarian metastasis, with AUCs of 0.54 (95% CI, 0.45–0.64) and 0.66 (95% CI, 0.56–0.77), respectively. When including CA125 in the model, the performance in discriminating between stage II–IV OC and stage I OC and between stage II–IV OC ovarian metastasis was improved (AUC increased from 0.88 to 0.94, *P* = 0.01, and from 0.86 to 0.97, *p* = 0.01).

**Conclusions:**

The IOTA ADNEX model has excellent performance in differentiating benign and malignant adnexal masses in the hands of nonexpert ultrasonographers with limited experience in China. In classifying different subtypes of ovarian cancers, the model has difficulty differentiating BOTs from stage I OC and BOTs from ovarian metastases.

## Introduction

In Chinese women, the mortality rates of breast cancer, cervical cancer and ovarian cancer are increasing year by year [1]. In particular, most ovarian cancer patients are asymptomatic in the early stage. The five-year survival rate of patients with stage III-IV ovarian cancer is less than 30%, that of patients with stage II ovarian cancer is approximately 70%, and that of patients with stage I ovarian cancer is more than 90% [2]. The combination of early diagnosis and timely treatment is considered to be the key factor to optimize the survival rate [3, 4]. The incorrect diagnosis of ovarian cancer as a benign tumour may delay the timing of treatment and lead to inadequate treatment; on the other hand, the incorrect diagnosis of a benign tumour as ovarian cancer can make patients undergo more extensive treatment and increase the possibility of postoperative complications. Thus, it is essential to make a correct diagnosis.

The diagnosis of adnexal masses mostly depends on ultrasonography. Some studies have reported that the subjective evaluation of a tumour by an expert ultrasonographer is an excellent method for discriminating between benign and malignant adnexal masses [5–7]. It is necessary for doctors who are not so experienced to use a more objective method to assist in diagnosis. To characterize ovarian tumours as benign or malignant, biomarkers combined with ultrasonography have been used to optimize the accuracy of diagnosis, including the risk of malignancy index (RMI). The International Ovarian Tumour Analysis (IOTA) group has presented a consensus on the terms, definitions and measurements used to describe the sonographic features of adnexal tumours [8] and standardized the description of ovarian lesions. Then, the IOTA developed and validated many models to discriminate between benign and malignant adnexal masses, such as the logistic regression models LR1 and LR2 and simple rules [9, 10]. In a meta-analysis [11], the ability of different methods to differentiate benign from malignant adnexal masses was compared. The results showed that the IOTA simple rules and LR2 were superior to RMI and to all other methods included in the meta-analysis.

The Assessment of Different NEoplasias in the adneXa (ADNEX) model is the first predictive multiclass model developed by the IOTA and is able to differentiate between benign tumours, borderline ovarian tumours (BOTs), stage I ovarian cancer (OC), stage II-IV OC and secondary metastatic ovarian cancers [12]. The preoperative characterization of an adnexal mass is crucial for selecting the optimal management strategy, and differential diagnosis of the mass by the ADNEX model may help to optimize management. In recent years, several studies have reported that the model has good to excellent performance in their populations [13–15]. Additionally, in China, this model has been reported to have high accuracy in distinguishing between benign and malignant adnexal masses by expert ultrasonographers in a gynaecological oncology centre in Shanghai [16]. However, there are few studies validating the discriminative performance of the ADNEX model in the hands of nonexpert ultrasonographers, and it has great potential as a method for the correct classification of adnexal masses by ultrasonographers with limited experience.

The aim of our study was to evaluate the performance of the IOTA ADNEX model in the preoperative discrimination of benign, borderline, early and advanced stage invasive, and secondary metastatic tumours in the hands of nonexpert ultrasonographers in a single oncology centre in Beijing, China.

## Methods

### Study design and patients

This was a single-centre diagnostic accuracy retrospective study conducted at a tertiary referral oncology hospital. From 1 January 2018 to 31 December 2019, 768 patients with an ultrasound diagnosis of an adnexal mass were consecutively recruited from the Department of Ultrasound in Beijing Obstetrics and Gynaecology Hospital in China.

The inclusion criteria were as follows: (1) the patients presented with at least one adnexal mass and underwent transvaginal or transrectal ultrasonography (supplemented with transabdominal ultrasonography if transvaginal ultrasonography is not sufficient); (2) the interval between operation and ultrasonography did not exceed 120 days; and (3) the patients had no previous history of ovarian cancer. The exclusion criteria were as follows: (1) cysts that were deemed to be clearly physiological and less than 3 cm in maximum diameter; and (2) previous bilateral adnexectomy. For bilateral adnexal masses, the mass with the most complex ultrasound features was included. If two masses had similar ultrasound morphologies, the largest mass or the one most easily accessible by ultrasonography was included [17]. The study was approved by the Institutional Ethics Committee of Beijing Obstetrics and Gynecology Hospital Affiliated to Capital Medical University.

Two nonexpert ultrasonographers at level 2 according to the EFSUMB classification who successfully passed the IOTA certification test exam assessed the sonographic tumour morphology based on the standardized manner previously published by the IOTA group [8]. All assessments were performed prior to obtaining pathology results, and the ultrasonographers were blinded to this outcome. The ultrasound machine used was a Voluson E8 system (GE Healthcare, USA) with 5.0–9.0 MHz transvaginal probes and 1.0–5.0 MHz transabdominal probes.

Clinical and ultrasound variables of the ADNEX model were recorded. Serum CA125 (U/ml) levels were assessed 7 days before surgery using Elecsys and Cobas E analysers (Roche, Mannheim, Germany).

### Reference standard

The histopathological diagnosis of the mass after surgical removal by laparoscopy or laparotomy was used as a reference standard. Tumours were staged according to the World Health Organization (WHO) classification of tumours, and malignant tumours were staged using the International Federation of Obstetrics and Gynecology (FIGO) standards [18]. In the final diagnosis, the masses were divided into five types: benign ovarian tumours, BOTs, stage I OC, stage II-IV OC, and secondary metastatic cancer (Table 1).

**Table 1:** Histopathological findings in 620 women with adnexal masses

Histological type	n (%)
Benign	402 (64.8)
Serous cystadenoma	115 (18.5)
Teratoma	111 (17.9)
Mucinous cystadenoma	81 (13.1)
Endometrioma	55 (8.9)
Fibrothecoma	15 (2.4)
Fibroma	7 (1.1)
Adenofibroma	2 (0.3)
Cystadenofibroma	2 (0.3)
Paraovarian cyst	6 (1.0)
Mesosalpinx cyst	4 (0.6)
Other benign ovarian lesion	4 (0.6)
Borderline	86 (13.9)
Serous	33 (5.3)
Mucinous	37 (6.0)
Endometrioid	2 (0.3)
Clear-cell	1 (0.2)
Sex cord-stromal tumours	13 (2.1)
Primary malignant	128 (20.6)
Serous adenocarcinoma	40 (6.5)
Clear cell carcinoma	31 (4.8)
Mucinous adenocarcinoma	28 (4.5)
Endometrioid adenocarcinoma	13 (2.1)
Serous/mucinous adenocarcinoma	7 (1.1)
Carcinosarcoma	3 (0.5)
Immature teratoma	3 (0.5)
Granulosa-cell tumour	2 (0.3)
Sertoli-Leydig	1 (0.2)
Ovarian metastasis	4 (0.6)

### ADNEX model

We input the variables needed for the ADNEX model into the web application (http://www.iotagroup.org/adnexmodel/). The model includes nine variables: age (years), serum CA125 level (U/mL), type of centre (oncology referral centre vs. non-oncology centre), maximal diameter of the lesion (mm), maximal diameter of the largest solid part (mm), number of papillary projections (0, 1, 2, 3 or more than 3), number of cyst locules (≤10 vs. > 10), acoustic shadows (yes or no), and ascites (yes or no) [12]. All ADNEX model parameters were logged objectively. Then, the model can calculate the patient-specific risk and relative risk of each subtype. With or without CA125 results, the model was able to calculate the malignant risk. This study compared the diagnostic accuracy of the model with or without CA125 results.

### Statistical analysis

We analysed data using R software. For statistical purposes, BOTs were considered malignant.

We compared the clinical and sonographic features of adnexal masses included in the ADNEX model using the chi-square test and Fisher’s exact test for categorical data and the Mann–Whitney U-test for continuous data. To validate the ADNEX model with and without CA125 levels, receiver operating characteristic (ROC) curve analysis was performed. We calculated the area under the curve (AUC) with 95% CIs for basic discrimination between benign and malignant adnexal tumours using the total risk of malignancy (i.e., the sum of the estimated risks of the four malignant subtypes). The AUCs of the ADNEX model with and without CA125 levels were computed for each pair of tumour types using the DeLong test.

We calculated the sensitivity, specificity, positive predictive value (PPV), negative predictive value (NPV), positive likelihood ratio (LR+) and negative likelihood ratio (LR-) at progressive cut-off points for the total risk of malignancy and at the cut-off point determined by ROC curve analysis of our data.

Statistical calculations were performed using 95% CIs, with *P* < 0.05 considered significant.

## Results

**Fig. 1:**
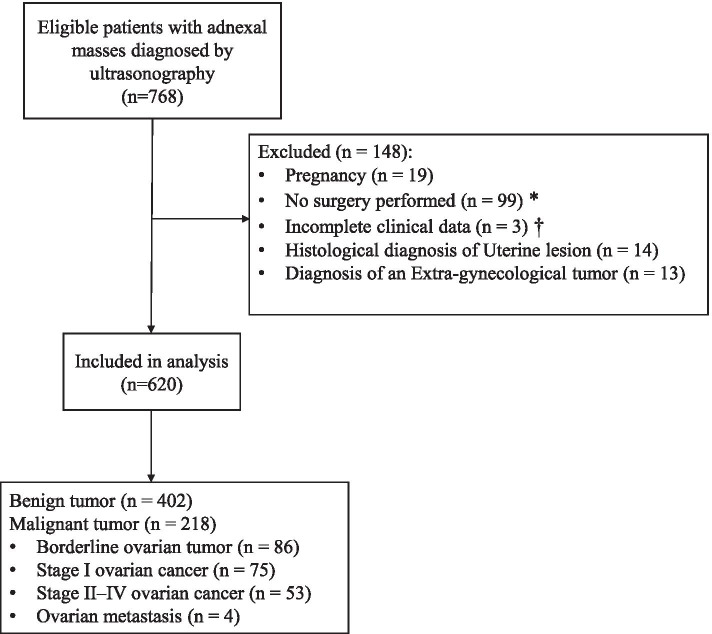
Flowchart showing the enrolment of women with adnexal mass and reasons for exclusion. *No surgery was performed in 99 patients because surgery was delayed due to neoadjuvant chemotherapy (*n* = 34), patients were in poor physical condition and unable to accept surgical treatment (*n* = 37), and patients decided not to undergo the operation for personal reasons (*n* = 28). † Incomplete clinical data refers to missing CA125 levels

Between 1 January 2018 and 31 December 2019, 768 patients with adnexal tumours were examined by ultrasonography before laparoscopy or laparotomy. A total of 148 women were excluded from the study because of pregnancy, failure to undergo surgery, incomplete clinical data, histological diagnosis of uterine lesion, or diagnosis of an extragynaecological tumour. Therefore, the final cohort consisted of 620 patients (Fig. 1).

Among them, 402 (64.8%) had benign tumours and 218 (35.2%) had malignant tumours, including 86 (13.9%) with BOT, 75 (12.1%) with stage I OC, 53 (8.5%) with stage II-IV OC, and 4 (0.6%) with ovarian metastases. The most common benign tumours were serous cystadenoma and teratoma, while the most common malignant tumours were serous adenocarcinoma and clear cell carcinoma.

The clinical and sonographic features of adnexal masses in our cohort are shown in Table 2. The patients in the malignant group were older and had higher CA125 levels than those in the benign group. The prevalence of solid tissue, papillary projections and ascites was more common in the malignant group. Acoustic shadows were more common in the benign tumour group. In addition, the prevalence of features including the maximum diameter of the lesion and the largest solid component, more than 10 locules and the presence of ascites were significantly different between benign and malignant masses (*p* < 0.05).

**Table 2:** Sonographic features of tumours in 620 women with adnexal masses

Characteristic	Malignant (*n* = 218)						
	Benign (*n* = 402)	Borderline (*n* = 86)	Stage I OC (*n* = 75)	Stage II–IV OC (*n* = 53)	Metastasis (*n* = 4)	Total (*n* = 620)	*p*
Age (years)	31 (27–39)	38 (30–48)	47 (41–53)	48 (44–57)	57 (46–62)	44 (34–52)	< 0.001*
CA 125 (U/mL)	11.4 (8–17)	18 (10–28)	37 (15–83)	204 (53–547)	66 (28–137)	26 (13–74)	< 0.001 *
Max diameter of lesion (mm)	63 (50–83)	88 (53–121)	106 (71–148)	88 (64–143)	81 (63–108)	92 (64–133)	< 0.001*
Presence of solid tissue	44 (10.9)	63 (73.3)	70 (93.3)	53 (100)	4 (100)	190 (30.6)	< 0.001 †
Maximum diameter of largest solid component, if present (mm)	30 (13–48)	31 (21–49)	46 (31–80)	66 (57–79)	74 (48–107)	45 (26–67)	*p* = 0.001*
Papillary projections present	15 (3.7)	45 (52.3)	36 (48.0)	28 (52.8)	0 (0)	109 (17.6)	< 0.001‡
0	387 (96.3)	41 (47.7)	39 (52.0)	25 (47.2)	4 (100)	109 (17.6)	
1	11 (2.7)	31 (36.0)	23 (30.7)	8 (15.1)	0 (0)	62 (10.0)	
2	2 (0.5)	7 (8.1)	3 (4.0)	4 (7.5)	0 (0)	14 (2.3)	
3	1 (0.2)	3 (3.5)	4 (5.3)	5 (9.4)	0 (0)	12 (1.9)	
> 3	1 (0.2)	4 (4.7)	6 (8.0)	11 (20.8)	0 (0)	21 (3.4)	
> 10 cyst locules	3 (0.7)	15 (17.4)	8 (10.7)	4 (7.5)	0 (0)	27 (4.4)	< 0.001 †
Acoustic shadows	121 (30.1)	1 (1.2)	5 (6.7)	2 (3.8)	0 (0)	8 (1.3)	< 0.001 †
Ascites	3 (0.7)	4 (4.7)	11 (14.7)	30 (56.6)	3 (75.0)	48 (7.7)	< 0.001 †

Data are given as the median (interquartile range) or n (%)

*OC* Ovarian cancer

P for benign vs. malignant group calculated using the following tests: *Mann–Whitney U-test, †chi-square test or ‡Fisher’s exact test

### Validation of the IOTA ADNEX model

**Fig. 2:**
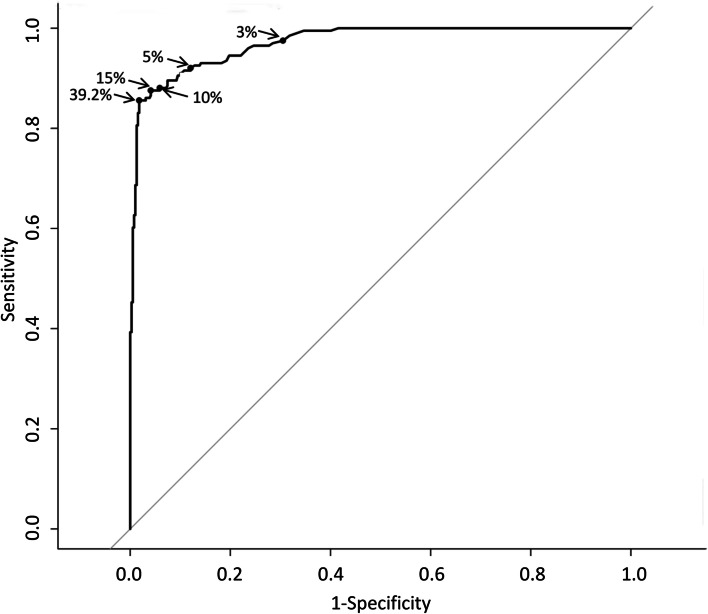
Receiver operating characteristic (ROC) curves for the performance of the International Ovarian Tumor Analysis ADNEX model in discriminating between benign and malignant adnexal masses. The optimal cut-off (the maximum value of the Youden index) was 39.2% for the probability of malignancy, at which the sensitivity was 87.06%, specificity was 97.69%, positive predictive value was 95.03%, negative predictive value was 93.66% and area under the ROC curve was 0.925. Cut-offs of 3.0, 5.0, 10.0 and 15.0% are also indicated

The diagnostic performance of the IOTA ADNEX model is presented in Fig. 2. The AUC of the model to differentiate benign and malignant adnexal masses was 0.97 (95% CI, 0.96–0.98).

The performance outcomes of the IOTA ADNEX model with CA125 level at progressive cut-off points for the probability of malignancy are shown in Table 3. The sensitivity was 87.06% (82.09–93.03) and the specificity was 97.69% (91.03–99.23) at an optimal cut-off of 39.2% probability of malignancy.

**Table 3:** Performance of the ADNEX model in discriminating between benign and malignant tumours at progressive cut-offs for probability of malignancy

Cut-off	AUC (95% CI)	Sensitivity (95% CI) (%)	Specificity (95% CI) (%)	PPV (95% CI) (%)	NPV (95% CI) (%)	LR+ (95% CI)	LR- (95% CI)	DOR
3%	–	97.51 (95.02–99.50)	69.49 (64.87–74.10)	62.22 (58.70–66.01)	98.22 (96.55–99.63)	3.20 (2.75–3.72)	0.04 (0.02–0.09)	80.00
5%	–	92.04 (88.05–95.52)	87.95 (84.62–91.03)	79.83 (75.30–84.16)	95.54 (93.33–97.49)	7.64 (5.82–10.02)	0.09 (0.06–0.15)	84.89
10%	–	88.06 (83.58–92.54)	94.10 (91.79–96.41)	88.61 (84.54–92.57)	93.92 (91.71–96.05)	14.93 (10.01–22.26)	0.13 (0.09–0.18)	114.85
**15%**	–	87.56 (82.59–92.04)	95.90 (93.85–97.69)	91.75 (88.02–95.31)	93.75 (91.57–95.84)	21.36 (13.18–34.61)	0.13 (0.09–0.19)	164.31
**39.2%**^a^	0.97 (0.96–0.98)	87.06 (82.09–93.03)	97.69 (91.03–99.23)	95.03 (84.07–98.35)	93.66 (91.41–96.24)	37.69 (18.09–78.53)	0.13 (0.09–0.19)	289.92

*AUC* Area under the receiver operating characteristic curve, *DOR* Diagnostic odds ratio, *LR+* Positive likelihood ratio, *LR–* Negative likelihood ratio, *NPV* Negative predictive value, *PPV* Positive predictive value

^a^Optimal cut-off, the maximum value of the Youden index

When tumours were classified into benign, BOTs, stage I OC, stage II-IV OC, and secondary metastatic cancer, the model showed poor to excellent discrimination between the different subtypes, with AUCs varying between 0.54 and 0.99 when the CA125 level was included in the model and between 0.50 and 0.99 without the CA125 level (Table 4). The AUCs of the model in differentiating benign tumours from subtypes of malignant tumours were high. The AUC was 0.94 for differentiating benign tumours from borderline tumours, 0.98 for differentiating benign tumours from stage I OC, 0.99 for differentiating benign tumours from stage II-IV OC, and 0.99 for differentiating benign tumours from secondary metastatic cancer. The ability to discriminate between benign and stage II–IV tumours and benign and secondary metastatic tumours was near perfect for the model with and without CA125 (AUC 0.99). In comparison, the model had more difficulties discriminating between borderline and stage I tumours (AUC 0.54) and between borderline and secondary metastatic tumours (AUC 0.66). It was able to distinguish stage II-IV cancer from other malignancies (AUC for stage II-IV cancer versus borderline tumours was 0.92, versus stage I cancer was 0.94, and versus secondary metastatic cancer was 0.97).

**Table 4:** Performance of the ADNEX model in polytomous discriminations between different types of adnexal masses according to whether CA 125 level was included in the model

Discrimination	AUC (95% CI)		*P*
	ADNEX model with CA 125	ADNEX model without CA 125	
Benign vs. Malignant	0.97 (0.96–0.98)	0.97 (0.95–0.98)	0.07
Benign vs. BOT	0.94 (0.92–0.97)	0.94 (0.91–0.97)	0.19
Benign vs. Stage I OC	0.98 (0.97–0.99)	0.98 (0.96–0.99)	0.21
Benign vs. Stage II–IV OC	0.99 (0.99–1.00)	0.99 (0.99–1.00)	0.03
Benign vs. Metastasis	0.99 (0.98–1.00)	0.99 (0.97–1.00)	0.24
BOT vs. Stage I OC	0.54 (0.45–0.64)	0.50 (0.41–0.60)	0.10
BOT vs. Stage II–IV OC	0.92 (0.88–0.97)	0.89 (0.88–0.97)	0.06
BOT vs. Metastasis	0.66 (0.56–0.77)	0.52 (0.29–0.75)	0.34
Stage I OC vs. Stage II–IV OC	0.94 (0.88–0.99)	0.88 (0.80–0.96)	0.01
Stage I OC vs. Metastasis	0.72 (0.60–0.85)	0.54 (0.21–0.86)	0.37
Stage II–IV OC vs. Metastasis	0.97 (0.93–1.00)	0.86 (0.76–0.95)	0.01

Comparison of the area under the receiver operating characteristic curve (AUC) of the ADNEX model with vs. without the inclusion of CA 125 level using the DeLong test

*BOT* Borderline ovarian tumour, *OC* Ovarian cancer

When including CA125 in the model, the performance in discriminating between stage II–IV OC and stage I OC and between stage II–IV OC and secondary metastatic tumours was improved (Tables 4 and 5). The validation AUCs increased from 0.88 to 0.94, *p* = 0.01 (stage II-IV OC vs. metastatic cancer) and from 0.86 to 0.97, *p* = 0.01 (stage II-IV OC vs. stage I OC).

**Table 5:** Performance of the ADNEX model with vs. without CA 125 level in discriminating stage I OC vs. stage II–IV OC and stage II–IV OC vs. metastasis

ADNEX model	AUC (95% CI)	Sensitivity (95% CI) (%)	Specificity (95% CI) (%)	PPV (95% CI) (%)	NPV (95% CI) (%)	LR+ (95% CI)	LR- (95% CI)	DOR	^a^Optimal cut-off (%)	*P*
Benign vs. Stage II–IV OC										
With CA 125	0.99 (0.99–1.00)	100.00 (100.00–100.00)	98.97 (97.44–100.00)	92.73 (83.61–100.00)	100.00 (100.00–100.00)	97.09 (43.80–215.22)	0.00 (−)	0.00	42.95	0.03
**Without CA 125**	0.99 (0.99–1.00)	100.00 (100.00–100.00)	97.69 (96.15–99.23)	85.00 (77.27–94.44)	100.00 (100.00–100.00)	43.29 (22.70–82.57)	0.00 (−)	0.00	40.75	
Stage I OC vs. Stage II–IV OC										
With CA 125	0.88 (0.80–0.96)	80.39 (68.63–90.20)	98.53 (95.59–100.00)	97.67 (92.68–100.00)	87.01 (80.95–93.15)	54.69 (7.78–384.48)	0.20 (0.11–0.35)	273.45	36.2	0.01
**Without CA 125**	0.94 (0.88–0.99)	84.31 (72.55–94.12)	98.53 (95.59–100.00)	97.78 (93.18–100.00)	89.33 (82.93–95.65)	57.35 (8.17–402.77)	0.16 (0.08–0.30)	358.44	30.55	
Stage II–IV OC vs. metastasis										
With CA 125	0.86 (0.76–0.95)	100.00 (100.00–100.00)	84.31 (72.55–92.16)	33.33 (22.22–50.00)	100.00 (100.00–100.00)	6.37 (3.50–11.61)	0.00 (−)	–	31.25	0.01
Without CA 125	0.97 (0.93–1.00)	100.00 (100.00–100.00)	96.08 (90.20–100.00)	66.67 (44.44–100.00)	100.00 (100.00–100.00)	25.51 (6.56–99.24)	0.00 (−)	–	15.2	

Comparison of AUC of ADNEX model with vs. without the inclusion of CA 125 level using the DeLong test

*AUC* Area under the receiver operating characteristic curve, *DOR* Diagnostic odds ratio, *LR+* Positive likelihood ratio, *LR–* Negative likelihood ratio, *NPV* Negative predictive value, *OC* Ovarian cancer, *PPV* Positive predictive value

^a^Optimal cut-off, the maximum value of the Youden index

## Discussion

In our study, we found that in the hands of nonexpert ultrasonographers with limited experience, the IOTA ADNEX model can distinguish benign and malignant masses, and its performance is similar to that achieved by experienced ultrasonographers in the original ADNEX validation study published by the IOTA team [12]. Regardless of whether the CA125 level is included, the IOTA ADNEX model showed an excellent ability to distinguish benign and malignant masses in a Chinese oncology centre (AUCs of 0.97 with and without CA125). Our results are also consistent with those of another Chinese validation study in which the model was validated by expert ultrasonographers [16].

Except for BOTs vs stage I OC and BOTs vs ovarian metastases, the ADNEX model showed good to excellent performance in distinguishing most of the subtypes of adnexal masses in our study (AUCs ranged from 0.72 to 0.99), especially benign tumours vs stage II-IV OC (AUC 0.99), benign tumours vs ovarian metastases (AUC 0.99), BOTs vs stage II–IV OC (AUC 0.92), stage I OC vs stage II–IV OC (AUC 0.94) and stage II–IV OC vs ovarian metastases (AUC 0.97), which were consistent with the results of other studies [13, 14, 16, 19]. However, the prediction of specific subtypes of malignant tumours had lower performance. When discriminating between BOTs and stage I OC and between borderline and secondary metastatic tumours, the AUCs were 0.54 and 0.66, respectively, which are both lower than previous research results [13, 14, 16, 19]. There are many overlapping features between BOTs and OC, especially early-stage OC, so it is very challenging to differentiate them in clinical practice. The survival rate of patients with borderline ovarian tumours confined to the ovary is high, almost 100% within 10 years [20]. BOTs often affect young women, and one-third of them are diagnosed under 40 years old, so fertility-preserving therapy should be considered [21]. A meta-analysis showed that women with early OC who underwent laparoscopic surgery had a lower incidence of complications and no significant difference in recurrence rates compared with those who underwent laparotomy [22]. For nonexpert ultrasonographers with limited experience, the ADNEX model can help identify the subtypes of ovarian tumours, except BOTs vs. stage I OC and BOTs vs. ovarian metastases.

In our validation study, using a 15% cut-off value to define malignancy, the ADNEX model achieved 87.6% sensitivity and 95.9% specificity, compared with 94.5 and 78.7% in the original study [12]. Although the sensitivity decreased, the specificity increased significantly, which helps to reduce the misdiagnosis rate of noncancer patients. In our clinical practice, we can choose the appropriate cut-off value according to the needs. According to the IOTA group study results, a 10% risk cut-off for the ADNEX model is recommended for non-oncological centres. However, because of the much higher percentage of malignant cases operated on in oncology centres, we used a much higher probability cut-off level (i.e., 37%) in this study. In our population, the IOTA ADNEX model had high positive and negative predictive values, which were slightly higher than those in other validation studies [14, 15]; thus, it could be considered an appropriate method for differentiating benign and malignant ovarian tumours in China.

The ADNEX model can make a more personalized diagnosis of ovarian tumours by identifying the types of malignant tumours (borderline, primary stage I, primary II- stage IV or secondary metastatic). This model can help clinicians choose the right treatment, choose conservative treatment, or plan the most appropriate surgical procedure (laparoscopic or open surgery) when surgery is needed or prompt doctors to find the primary site of the tumour when masses are assessed as metastatic cancer. We have shown that the ADNEX model performs equally well in the hands of nonexpert ultrasonographers with limited experience compared to the initial study, but the differential diagnosis between BOTs and stage I OC and BOTs and ovarian metastases needs to be improved.

### Strengths and weaknesses

The main advantage of our study is that it is the first validation study in the hands of nonexpert ultrasonographers with limited experience in China. The researchers successfully passed the IOTA certification test, so tumour morphology could be evaluated in strict accordance with the IOTA consensus statement while blinded to the pathology results. Every patient in our centre had a preoperative CA125 measurement using the same methodology.

The limitation of our study is that it is a retrospective study, which might have introduced selection bias. There are fewer cases of ovarian metastatic cancer, which cannot guarantee that the ADNEX model can draw reliable conclusions when distinguishing it from other subtypes.

## Conclusions

The IOTA ADNEX model has excellent performance in differentiating benign and malignant adnexal masses in the hands of nonexpert ultrasonographers with limited experience in China. In classifying different subtypes of ovarian cancers, the model has difficulty differentiating BOTs from stage I OC and BOTs from ovarian metastases.

## Data Availability

The dataset supporting the conclusions of this article is included within the article and its additional files.

## Abbreviations

IOTA: International Ovarian Tumor Analysis
ADNEX model: Assessment of Different NEoplasias in the adneXa model
BOT: Borderline ovarian tumour
OC: Ovarian cancer
ROC curve: Receiver operating characteristic curve
AUC: Area under the curve
CA 125: Cancer antigen 125
PPV: Positive predictive value
NPV: Negative predictive value
LR+: Positive likelihood ratio
LR-: Negative likelihood ratio
